# Do Competing Demands Elevate Caregiver Challenges?

**DOI:** 10.1093/geroni/igaa057.2193

**Published:** 2020-12-16

**Authors:** Rosemary Blieszner, Tina Savla, Karen Roberto, Brandy Renee McCann, Aubrey Knight, Emily Hoyt

**Affiliations:** 1 Virginia Tech, Blacksburg, Virginia, United States; 2 Virginia Polytechnic Institute and State University, Blacksburg, Virginia, United States; 3 Virginia Tech Carilion School of Medicine, Roanoke, Virginia, United States

## Abstract

Family members caring for relatives with dementia simultaneously hold other roles and face other everyday challenges related to employment, finances, interactions with others, and the like. Using the Pearlin stress process model as a foundation, we evaluated contributions of secondary role and intrapsychic stressors such as health, relationship, and financial worries, and role captivity and overload, to 157 rural caregivers’ morale and well-being. Whereas family conflict and role overload contributed significantly to higher agitation, money worries, poor health, and role captivity interfered with positive mental health (p<.05). Nevertheless, some caregivers characterized employment and volunteering as respite from caregiving and appreciated informal help even when family ties were complicated. Findings highlight complex responses to dementia-related caregiving and point to the need for a range of resources and support deployed creatively to assist caregivers in places such as rural Appalachia where geography, underfunding, and low income limit access to services.

